# Implicit Learning, Bilingualism, and Dyslexia: Insights From a Study Assessing AGL With a Modified Simon Task

**DOI:** 10.3389/fpsyg.2019.01647

**Published:** 2019-07-26

**Authors:** Maria Vender, Diego Gabriel Krivochen, Beth Phillips, Douglas Saddy, Denis Delfitto

**Affiliations:** ^1^Department of Cultures and Civilizations, University of Verona, Verona, Italy; ^2^Centre for Integrative Neuroscience and Neurodynamics, University of Reading, Reading, United Kingdom

**Keywords:** artificial grammar learning, implicit learning, bilingualism, dyslexia, bilingualism and dyslexia interaction

## Abstract

This paper presents an experimental study investigating artificial grammar learning in monolingual and bilingual children, with and without dyslexia, using an original methodology. We administered a serial reaction time task, in the form of a modified Simon task, in which the sequence of the stimuli was manipulated according to the rules of a simple Lindenmayer grammar (more specifically, a Fibonacci grammar). By ensuring that the subjects focused on the correct response execution at the motor stage in presence of congruent or incongruent visual stimuli, we could meet the two fundamental criteria for implicit learning: the absence of an intention to learn and the lack of awareness at the level of resulting knowledge. The participants of our studies were four groups of 10-year-old children: 30 Italian monolingual typically developing children, 30 bilingual typically developing children with Italian L2, 24 Italian monolingual dyslexic children, and 24 bilingual dyslexic children with Italian L2. Participants were administered the modified Simon task developed according to the rules of the Fibonacci grammar and tested with respect to the implicit learning of three regularities: (i) a red is followed by a blue, (ii) a sequence of two blues is followed by a red, and (iii) a blue can be followed either by a red or by a blue. Results clearly support the hypothesis that learning took place, since participants of all groups became increasingly sensitive to the structure of the input, implicitly learning the sequence of the trials and thus appropriately predicting the occurrence of the relevant items, as manifested by faster reaction times in predictable trials. Moreover, group differences were found, with bilinguals being overall faster than monolinguals and dyslexics less accurate than controls. Finally, an advantage of bilingualism in dyslexia was found, with bilingual dyslexics performing consistently better than monolingual dyslexics and, in some conditions, at the level of the two control groups. These results are taken to suggest that bilingualism should be supported also among linguistically impaired individuals.

## Introduction

The extent to which bilingualism can enhance executive functions (EFs) as well as metalinguistic skills (Bialystok et al., 2008, 2014) attracts vast research interest. However, a sparse number of studies has explored the interaction between bilingualism and atypical development, in order to investigate whether these advantages extend also to individuals suffering from specific impairments such as developmental dyslexia^1^. This would have a crucial social impact, since parents and teachers of impaired children often fear that bilingualism could negatively affect their linguistic development and could thus decide that one of the languages should be abandoned (Vender et al., 2018a; Garaffa et al., 2019).

Importantly, the limited available evidence seems to suggest that the positive effects associated to bilingualism in metalinguistic tasks not only extend also to bilingual children with dyslexia, but can be even more marked than in typical populations [see Vender et al. (2018b) for a study on nonword pluralization]. Conversely, the relationship between bilingualism and dyslexia in the domains of EF and implicit learning has not been examined yet.

With the aim of bridging this gap, we investigated the interaction between these two populations (bilingual and dyslexic children) in a task assessing implicit learning, using a modified Simon task in which the sequence of the stimuli is determined by the rules of an artificial grammar.

This paper is organized as follows: we first introduce the concept of artificial grammar learning (AGL), reporting the studies assessing implicit learning in bilinguals as well as in dyslexic children especially focusing on the grammar that we employed in the present study, the Fibonacci grammar. We then discuss the literature addressing the performance of bilinguals and that of dyslexics in the Simon task and formulate our research questions and predictions. Finally, we present our experimental task discussing its results and implications.

### Bilingualism and Dyslexia: What Artificial Grammar Learning Can Tell Us

Artificial grammar learning is an experimental paradigm employed to investigate how sequences of symbols generated by a system are learnt. Once exposed to an artificial grammar (a set of rules that applies to an alphabet of symbols to generate strings), participants are assumed to develop some “implicit” knowledge of the regularities associated with it. In a typical AGL task, subjects first complete a training session in which they are exposed to stimuli arranged according to an invented grammar and are asked to pay attention to them, often by means of a recall task. After this training phase, they are made aware that these stimuli comply with a set of rules and are then instructed to provide grammaticality judgments for new sets of items which either are consistent with these rules (i.e., grammatical) or violate them (i.e., ungrammatical).

Results of classical AGL studies (e.g., Reber, 1967), which have been extensively replicated, indicate that people are successful in discriminating grammatical from ungrammatical stimuli, although they do not display conscious knowledge of the rules. These typically remain, at least in part, implicit [see Pothos (2007) for a general review of the different theoretical accounts of AGL performance]. The ability to detect patterns and statistical regularities in an artificial grammar has been found also in very young children (Gómez and Gerken, 2000). This capacity provides evidence for statistical learning based on transitional probabilities to compute distributional information and formulate relevant hypotheses about following stimuli (Saffran et al., 1996; Gerken et al., 2005). Moreover, it correlates with natural language learning and processing (Christiansen et al., 2012), indicating that AGL can provide a useful tool for investigating the ways in which humans perceive and process stimuli, as well as for understanding higher-order cognitive functions, including language (Pothos, 2007; De Vries et al., 2008). Therefore, AGL offers new ways to investigate specific aspects of language learning that are not easily testable with natural languages, such as analyzing language acquisition and processing, while also investigating the underpinnings of the human language faculty in a controlled setting (Ettlinger et al., 2016). Using language-independent rules (which nonetheless share properties with the kind of computational devices that are hypothesized to underlie grammatical competence) and non-linguistic stimuli has several practical advantages in implicit learning paradigms: in particular, it allows speakers of different native languages to be compared across one medium (Culbertson et al., 2013); it allows young children who may not have fully acquired language as well as nonverbal populations to be tested on that medium (Gomez and Gerken, 1999); and it allows researchers to fine-tune the paradigm with a precision that is limited only by their understanding of the mathematical properties of the rules and the structures thereby generated.

There are other notable methodological benefits: the participant has not been exposed to the stimulus beforehand, so observed experimental effects can be reliably linked back to the grammar, and implicit learning can be observed independently of factors which play a major role in the natural language parsing, such as semantics and pragmatics (Lobina, 2011). More particularly, it is possible to isolate specific local units for analysis without worrying about confounding factors related to the content of the symbols being used.

Artificial grammar learning has, more recently, been used to explore implicit learning in atypical populations, including individuals suffering from language-related impairments, such as aphasia (Christiansen et al., 2010) and developmental language disorder/specific language impairment (Evans et al., 2009). As for developmental dyslexia, deficits in AGL have been reported by Pavlidou and Williams (2014), who found that school-aged children with dyslexia showed difficulties in implicit learning; more specifically, in higher-order rule-like learning. Using a nonverbal task assessing AGL by presenting geometric shapes arranged either sequentially or in an embedded way, Pothos and Kirk (2004) found evidence for a different learning strategy in dyslexic adults in comparison to controls; impaired subjects were less skilled in processing the individual elements of the stimuli. Other studies confirmed that dyslexics are impaired in implicit learning tasks, indicating that they struggle in identifying and assimilating systematic patterns of stimuli in a structured setting, independently of the learning materials (Folia et al., 2008; Goldberg, 2014).

However, other studies have reported that dyslexics show no disadvantages in AGL (Rüsseler et al., 2006), which suggests that the complexity of the learning environment (in terms of processing costs) could play a major role (Vicari et al., 2005; Roodenrys and Dunn, 2007; Pavlidou et al., 2010; Nigro et al., 2015). Consistently, Katan et al. (2017) administered to the same group of children two AGL tasks differing in the type of grammar adopted, and found that children with dyslexia, although performing worse than controls with the grammars that, according to the authors, were more difficult to learn, showed intact learning of the less complex grammar, suggesting that they managed to extract relevant regularities from the input under less demanding conditions.

All in all, these results seem to suggest that dyslexics, despite exhibiting problems in the implicit detection and abstraction of rules under complex conditions, nevertheless do show a sensitivity to structural regularities in AGL (Pavlidou et al., 2010). Their difficulties could then be attributed to working memory (WM) restrictions: due to their limitations in WM and in processing capacity [see Nicolson and Fawcett (2008) and Vender (2017) for accounts based on processing deficits in dyslexia], dyslexics could be less efficient than their peers in formulating and simultaneously comparing different hypotheses depending on the structural regularities of the input (Baddeley, 1983).

Artificial grammar learning in bilingualism has not been extensively studied and the limited results available are mixed: Onnis et al. (2018) reported heightened performance in bilinguals in two AGL tasks while individual variables were controlled for; similarly, a bilingual advantage in statistical learning has been reported by other studies (Bartolotti et al., 2011; Escudero et al., 2016). Conversely, no differences were found by Yim and Rudoy (2013). Poepsel and Weiss (2016) compared monolingual and bilingual adults in a statistical word-learning task, reporting similar performance of the two groups with a moderate level of processing difficulty, but evidence for a bilingual advantage, with an increased level of processing difficulty, suggesting that basic statistical learning is not affected by bilingualism, whereas a bilingual advantage could arise in more complex tasks that require inhibiting potential sources of interference.

To summarize so far, the studies conducted until now have typically investigated AGL by explicitly exposing subjects to visually or auditorily presented sequences of symbols produced by a grammar, and explicitly asking subjects, after training, to provide acceptability judgments on these (or new) sequences of symbols. The results of these studies confirm that AGL takes place across different ages, measured by above-chance performance in the grammaticality tasks, in healthy subjects as well as in bilinguals, who in some cases have been found to outperform monolinguals. Although displaying intact learning in easier conditions, dyslexic subjects have instead been found impaired in conditions requiring more costly processing.

The present study investigates AGL in monolingual and bilingual children, with and without dyslexia, using a radically different methodology: instead of overtly training the subjects with sequences of symbols and asking for grammaticality judgments after training, we administered a serial reaction time (SRT) task; more specifically, a modified version of the Simon task. In our version, the sequence of visually presented stimuli is not random, but predictable on the basis of systematic regularities that characterize the output of the grammar we used. In this way, we can fully exploit the advantages of a SRT task in order to preserve the implicit nature of AGL. Under these experimental conditions, the two main requirements for implicit learning (i.e., absence of an intention to learn and lack of awareness of the acquired knowledge) are clearly guaranteed. This constitutes an original aspect of our protocol. Even more original is our use of a set of rules belonging to a class of grammars different from those used in traditional AGL experiments, as will be discussed below.

### The Fibonacci Grammar: A Simple Lindenmayer System

To date, AGL tasks have primarily used grammars in “canonical form” (Jäger and Rogers, 2012). These grammars, by definition, consist of (1) an alphabet which includes a start symbol (i.e., the symbol from which the rewriting procedure originates), rewriteable symbols (i.e., symbols which are written as other symbols, continuing the rewriting procedure), and non-rewriteable symbols (i.e., symbols that stop rewriting and correspond to the terminal forms of the strings generated) and (2) a set of rules of the form “rewrite A as B” which determine specifically how the grammar is developed by rewriting symbols in the alphabet in a stepwise manner, as will be described below. By applying these rewriting rules left-to-right sequentially to a set of symbols, grammatical “strings” are generated, also termed “words” or “sentences.” An example is the kind of phrase-structure rules familiar from linguistics, where → is simply “rewrite left-hand side as right-hand side” [i.e., “every time you find the symbol in the left in your input string, replace it with the symbol(s) in the right”], follows in (1):

(1) Sentence → Noun Phrase + Verb Phrase

The rule above encodes hierarchical constituency in a sentence: a symbol Sentence is rewritten as two non-terminals Noun Phrase (NP) and Verb Phrase (VP). Further structural details can be provided in the form of the rule in (2):

(2) Noun Phrase → Determiner + Noun

In (2), both “Determiner” and “Noun” are *terminal symbols*, insofar as they do not rewrite as any other symbol. It is worth emphasizing that the second rule can only apply if the first has applied already: otherwise there is no “NP” symbol to rewrite. This strict sequentiality and inherent order in rule application is usually referred to as a “traffic convention,” and it is a crucial property of phrase structure grammars.

In this respect, it should be emphasized that familiar systems of the kind that are customarily referred to in order to describe natural language structure, traditionally giving rise to the much-discussed Chomsky hierarchy Chomsky (1956), do not exhaust the landscape of rule-based formalisms.

**FIGURE 1:**
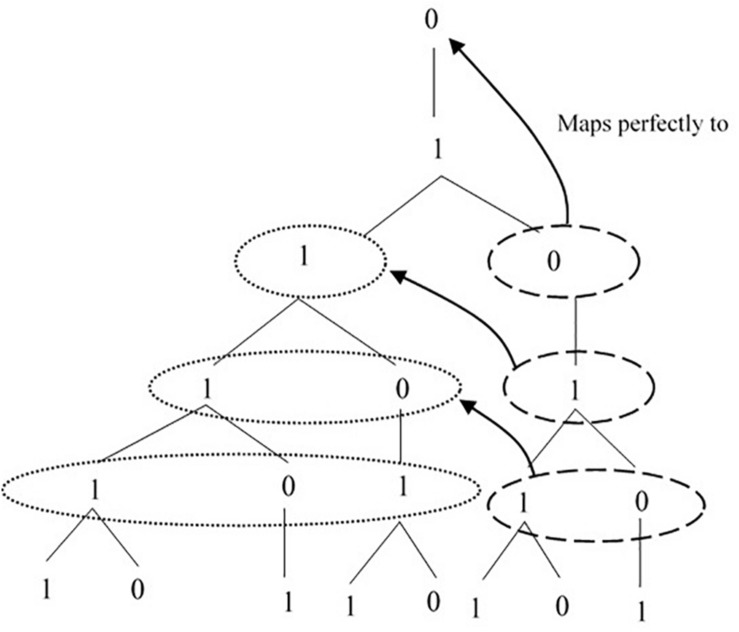
Self-similarity in the Fibonacci derivation.

Our implementation of AGL exploits one of these alternative formalisms: Lindenmayer systems. Lindenmayer grammars (Lindenmayer, 1968; Rozenberg and Salomaa, 1980; Prusinkiewicz and Lindenmayer, 2010) are simple deterministic recursive rewrite systems with some special properties. First, there is no distinction between nodes (nonterminals, i.e., symbols that are rewritten as other symbols; S, NP, and VP in the example above) and leaves (terminals, i.e., symbols that terminate the rewriting procedure; Determiner and Noun, above). Second, there is no “traffic convention,” indicating that all expandable symbols are effectively expanded *all at once*; expansion takes place in a top-down fashion, rather than left-to-right. Finally, they present self-similarity: each generation of the grammar maps to earlier generations, such that any natural constituent of the grammar can be used to reconstruct structural context, as displayed in Figure 1.

An important property of L-systems is that the strings that they generate contain a systematic range of statistical regularities. These follow from the formal properties of the grammar and can be controlled and probed for without *ad hoc* modifications. As a result, stimuli generated using L-systems provide an extraordinary platform for investigating the potential and limits of statistical learning (Saddy, 2009).

As argued above, previous research has shown that humans are able to extract information from signals, including natural and artificial grammars (Shirley, 2014; Geambasu et al., 2016; Phillips, 2017). However, identifying the specific kind of operation involved in this process is controversial. A non-randomly generated signal will present surface statistical regularities locally governing the transition between distinct symbols in the string, for whichever mode of presentation under consideration. It has been shown that these surface statistical effects can be found in children as young as 8 months old (Saffran et al., 1996) as well as in other species (e.g., Fehér et al., 2017). Given a signal, a fundamental question is whether statistical mechanisms are enough for an organism to infer or learn the underlying system of rules that has generated that signal and therefore make reliable hypotheses about adjacent and non-adjacent symbols in a sequence in locally ambiguous conditions. In this context, rule learning (which requires higher-order computational operations than the calculation of immediate transition probabilities in a string) has also been shown to be available very early on and to be essential for an adequate account of language and language-like phenomena (Marcus et al., 1999; Marcus and Berent, 2003).

For the purposes of the present paper, we have used a specific L-system, a so-called Fibonacci grammar (Fib grammar henceforth)^2^, defined by the following rules:

(3) 0 → 1
  1 → 1 0

The interpretation of such a formalism is very simple: every instance of [0] in a sequence must be replaced by (or “rewritten as”) [1], and every instance of [1] in the same sequence must be replaced by [1 0] in a top–bottom derivation. Applying these rules over and over again generates longer and longer sequences of symbols: specifically, the grammar in (3) generates derivations like the hierarchical sequence reported in Figure 1, where each row (a “generation” of the grammar) is a sequence of [1]s and [0]s and corresponds to a string of symbols. These strings of [1]s and [0]s can then be mapped onto linguistic or non-linguistic stimuli, across varying modalities.

An important derivational property of Fib-grammars [see Krivochen and Saddy (2018), Krivochen et al. (2018), Saddy (2018) for discussion about Fib grammars] is that each generation can be predicted if (i) we have access to the previous generation and to the rules, or (ii) we have access to two successive generations.^3^

The grammar presented in (3) generates strings in which the following first-order transitional regularities hold:

(4)
  (a) A [0] is always followed by a [1]
  (b) Two [1] are always followed by a [0]
  (c) A single [1] can be followed by either a [0] or a [1]

These regularities imply that the following *n*-grams are never to be found in the derivation of the Fib grammar, and are thus “ungrammatical”:

(4′) ^*^00
  ^*^111

In principle, (4c) could be seen as suggesting an element of non-determinism in the derivation of the grammar; but this is not so. The ambiguity that arises in single [1]s pertains *only* to left-to-right, very local transition probabilities: once we have more information about the string (i.e., if we have access to more symbols), these points can be disambiguated in a systematic way by reconstructing the underlying hierarchical structure (the “derivation”). In other words, simply by looking at its environment, we know without the need to reconstruct anything that if a [1] is preceded by another [1] the following symbol is [0]. If the [1] is preceded by a [0], instead, we face two possible scenarios, only one of which leads to a real ambiguity. The sequence […101**01**], indeed, is only apparently ambiguous, since it can be disambiguated by local structure reconstruction, i.e., going back one generation: since the previous generation of [1010] is [11], and since we know that [^*^111] is ungrammatical, we are forced to conclude that only a [1] can complete the sequence [10101]. The only case of real ambiguity presented by the Fibonacci grammar is found in the sequence [1101], since looking back to the string alone does not provide enough information to predict what comes, as it can be followed either by a [0] or by a [1]. Here, we will not go into further details regarding structural ambiguities in the Fib-string (see Krivochen et al., 2018), but it is important to be aware of these dependencies in order to understand the type of information that is being implicitly learned.

Given the properties illustrated above, a reasonable learning hypothesis is that there are two distinct processes going on at the same time: a low-level statistical process (“low level” because it is string-based), rooted in linear relations [see regularities (4a,c) above], and a high-level process rooted in the induction of relations between non-adjacent symbols (which require us to go beyond strictly linear relations, up to phrase-structure power).

### The Simon Task: Implications for Bilingualism and Dyslexia

In traditional versions of the Simon task (Simon, 1969), subjects are presented with random sequences of blue and red shapes appearing on the left or on the right side of a computer screen, and they are instructed to press distinct keys on the keyboard, depending on the color of the item only, ignoring its position on the screen. In “congruent” trials, the stimulus is on the same side as the key to be pressed, whereas in “incongruent” trials, the correct key is on the opposite side. Performance in terms of reaction times (RTs) and accuracy is typically worse (i.e., slower RTs and lower accuracy) for incongruent trials, which require more attentional resources in order to inhibit responses based on irrelevant information (i.e., the position of the square on the screen).

It has been found that bilinguals, across different ages, are more skilled than monolinguals in tasks tapping their EFs [see Adesope et al. (2010) for a review on 63 studies investigating EF in bilinguals; but see also Hilchey and Klein (2011) and Paap (2018) for a more critical perspective on the bilingual advantage], including the Simon task: bilinguals are indeed typically faster than monolinguals in this task, on both congruent and incongruent trials (Bialystok et al., 2004, 2005; Bialystok, 2006; Morton and Harper, 2007; Martin-Rhee and Bialystok, 2008).

As for an explanation for this advantage, no consensus has been reached yet. Some scholars have proposed that bilinguals display higher inhibitory control than monolinguals (Carlson and Meltzoff, 2008; Luk et al., 2010) or better EF in general (Bialystok et al., 2004): specifically, since their two (or more) languages are always active in the brain, they need to constantly inhibit the one which is not used at a given moment. This is suggested to make them generally more adept at focusing on relevant stimuli, inhibiting irrelevant ones. However, more recent studies have suggested that attentional control, instead of inhibition and interference suppression functions, is more enhanced in bilinguals. More particularly, Zhou and Krott (2018) hypothesized that bilinguals have greater abilities in engaging and maintaining vigilant attention in task performance: this allows them to avoid temporary lapses of attention which would lead to “temporary loss of task goals from the working memory” (p. 2). Crucially, enhanced attentional control leads to better performances in both conflict and non-conflict conditions, which would explain why bilinguals’ better performance in EF tasks, such as the Simon task, has been found not only in incongruent conditions, but also in congruent ones.

Conversely, EF is typically compromised in dyslexics, who display deficits in the maintenance of relevant information in WM, in both long-term memory access and retrieval and in the inhibition of irrelevant information [Varvara et al., 2014; see Booth et al. (2010) for a recent meta-analysis on children with reading disabilities].

In the present study, we tested learning of an artificial grammar by means of a modified Simon task. The paradigm was modified in two ways: (i) the sequence of stimuli was determined by the Fibonacci grammar (see section “The Fibonacci Grammar: A Simple Lindenmayer System”) instead of being “randomized,” and (ii) incongruent trials occurred at regular intervals (every sixth item). The first modification allowed us to verify whether statistical learning succeeds, manifesting itself in terms of faster RTs for predictable trials (corresponding to the unambiguous points in the series of visual stimuli as discussed above). The second modification, though less strictly tied to the experimental logic of the design, was implemented in order to limit the conflict between congruent and incongruent trials, by making incongruent trials regularly occurring and therefore statistically predictable. This conflict, which plays a central role in the traditional Simon task is, for the most part, devoid of interest for the purposes of the present experimental design. Introducing a regular repeat was expected to be sufficiently easy to maintain the nature of the Simon task while adding a simple regularity for subjects to detect. Furthermore, the occurrence of the incongruent trial every 6 was long enough to allow anticipation, so as to involve some limited kind of effort. This arguably contributed to keep the task engaging for the participants.

It should be emphasized that there are important advantages in adopting the Simon task, as a widely applied experimental tool in cognitive sciences. First of all, it allows direct targeting of subjects’ abilities to extract regularities from the input without conscious awareness. It also allows for the creation and presentation of stimuli which are visual instead of verbal, thus yielding a language-independent task. Furthermore, in a SRT task such as this, participants are only required to respond to visual stimuli (a challenge made relatively complex by the conflict between congruent and incongruent trials), to the effect that the participants’ conscious attention is arguably diverted from the patterns that these stimuli follow. More particularly, since the participants’ only concern is to respond correctly to the trials, the possibility that they take “chance” decisions is plausibly lower than in designs where they are requested to provide a grammaticality judgment, even when they feel unsure about the answer. This means that SRT paradigms are not required to meet the “zero correlation criterion” in order to observe truly implicit learning [see Dienes (2008) for a discussion about the verification of implicit learning in AGL experiments]. Evidence for implicit learning using a SRT task is provided by Cleeremans and McClelland (1991), indicating that this can offer a viable tool for assessing automatic learning of sequential material (see also Goldberg, 2014).

### Research Questions and Predictions

In light of what discussed above, we were first of all interested to establish whether there was any learning of the transitional rules of the Fibonacci grammar during the execution of our modified Simon task, supported by a decrease in RTs in the trials where the following stimulus was predictable on the basis of the transitional regularities induced by the grammar on the output. Since the Fib grammar is non-canonical, arguably instantiating some kind of more abstract and potentially language-independent grammatical knowledge, this result is of interest in itself.

Second, and more importantly, we were interested to establish whether, and to what extent, these learning effects also manifested themselves within the two populations in question (bilinguals and dyslexics), and whether there was, with respect to learning, any interaction between bilingualism and dyslexia.

As for dyslexia, the sparse studies on AGL involving a SRT task (Goldberg, 2014) suggest that dyslexics may show learning improvements comparable to typically developing controls, although differences could arise in conditions requiring higher processing costs, as discussed above. Moreover, if dyslexics are found to be delayed in their learning process in comparison to controls, this could support prior predictions that the kind of procedural knowledge involved in implicit learning is, at least to some extent, impaired in dyslexia.

As for bilingualism, although the previous results from AGL research, as seen above, are not homogeneous, we are inclined to believe that the reportedly enhanced ability of bilinguals to track distributional regularities of the input across associated representations in different languages might result in increased efficiency and flexibility in generally detecting regularities through analysis of the input (Weiss et al., 2015). Since the ability to track distributional properties in the input is most plausibly linked to unconscious procedural knowledge, it should be possible to address it with a task assessing implicit learning. Moreover, we emphasize that in the lively debate about the cognitive aspects of bi- and multilingualism [see Bialystok et al. (2008) and Costa et al. (2008) for studies reporting advantages of bilingualism; but see also Hilchey et al. (2015) and Paap et al. (2015) for more critical perspectives], the role of learning as such has received only modest attention. In the research presented here we explicitly face exactly this issue: the modified version of the Simon task that we propose here clearly provides implicit learning opportunities for the subjects.

In a nutshell, our experimental hypotheses are thus as follows: (i) we predict that, in the experimental protocol outlined here, learning should succeed for all groups, supporting the robustness of implicit learning effects in SRT paradigms and, crucially, for non-canonical grammars; (ii) we predict that differences among the three groups might also be found, with dyslexics exhibiting less efficient learning and bilinguals performing better, for the reasons just mentioned. As already emphasized, we are also particularly interested in the bilingualism/dyslexia interaction, in order to assess whether bilingualism has a positive or negative influence on the dyslexics’ performance at the level of implicit learning, and whether the possible benefits of bilingualism extend also to impaired children. Based on the limited results available mentioned above, (iii) we expect in fact that the benefits of bilingualism, related to bilinguals’ enhanced attentional skills and improved procedural learning skills, extend also to children with dyslexia.

## The Current Study

### Participants

Our experimental protocol was administered to 108 children divided in four groups: 30 Italian monolingual typically developing children (MC; mean age 10.0 years old, *SD* = 1.2), 30 bilingual typically developing children with Italian as an L2 (BC; mean age 10.2 years old, *SD* = 1.2), 24 Italian monolingual dyslexic children (MD; mean age 10.0 years old; *SD* = 1.3), and 24 bilingual dyslexic children (BD; mean age 10.4 years old, *SD* = 1.4).

All the monolingual children were native speakers of Italian, whereas Italian was the L2 of all the bilingual children.^4^ A questionnaire was administered to gather information about their exposure to the two languages, including Age of First Exposure (AFE) to Italian, Quantity of Exposure (QE) in Italian, Traditional and Cumulative Length of Exposure (TLE and CLE) to Italian.^5^ All subjects were active bilinguals using their L1 principally at home with parents and siblings and their L2 at school. The results of the questionnaire for the bilingual groups are reported in Table 1. No significant differences were found among the two groups concerning AFE [*t*(51) = 0.504, *p* = 0.518], QE [*t*(51) = 0.612, *p* = 0.543], TLE [*t*(51) = 0.621, *p* = 0.537], and CLE [*t*(51) = 0.534, *p* = 0.595].

**TABLE 1:** Means (*standard deviations*) of age of first exposure (AFE), quantity of exposure (QE), traditional length of exposure (TLE), and cumulative length of exposure (CLE) to Italian of the two bilingual groups.

	**AFE (in years)**	**QE (in percentage)**	**TLE (in years)**	**CLE (in years)**
BD	2.52	0.67	7.71	2.27
	(*2.30*)	(*0.14*)	(*2.20*)	(*0.80*)
BC	2.24	0.64	8.08	2.39
	(*1.81*)	(*0.13*)	(*2.10*)	(*0.75*)

*BD, bilingual dyslexics; BC, bilingual controls.*

Bilingual and monolingual children attended the same public schools and lived in the same areas in the north of Italy (Trento and Verona). Regarding the socio-economic status of the participants, we considered parental education, asking parents to provide information about their educational level: one point was attributed to primary education (i.e., primary and middle school), two for secondary education (i.e., high school), and three for higher education (i.e., university). Each subjects’ parental education score was calculated as the average of their parents’ scores. No statistically significant differences between the four groups were found [*F*(3,104) = 1.558, *p* = 0.204; see Table 2 for mean values of each group].

**TABLE 2:** Means (*standard deviations*) of the preliminary measures for each group.

	**Nonverbal Intelligence Raven^a^**	**Vocabulary PPVT-R^b^**	**Word reading speed^a^**	**Nonword reading speed^a^**	**Word reading accuracy^a^**	**Nonword reading accuracy^a^**	**Forward digit span**	**Backward digit span**	**Nonword repetition**	**Parental education**
BD	0.12	90.50	–2.13	–0.88	–2.71	–2.75	23.92	10.33	0.60	1.92
	(*0.58*)	(*0.13*)	(*2.18*)	(*1.45*)	(*1.32*)	(*1.10*)	(*3.47*)	(*3.59*)	(*0.13*)	(*0.42*)
MD	0.10	108.21	–3.75	–2.86	–2.20	–2.13	24.87	9.50	0.67	2.00
	(*0.75*)	(*13.18*)	(*2.73*)	(*2.58*)	(*1.80*)	(*1.42*)	(*3.69*)	(*4.23*)	(*0.12*)	(*0.25*)
BC	0.20	95.80	0.99	0.65	0.04	0.22	27.57	13.60	0.84	1.85
	(*0.83*)	(*13.50*)	(*0.77*)	(*0.68*)	(*0.95*)	(*0.80*)	(*6.63*)	(*5.24*)	(*0.11*)	(*0.33*)
MC	0.37	105.13	0.25	0.26	0.25	0.26	29.23	12.97	0.86	2.03
	(*0.74*)	(*9.09*)	(*0.87*)	(*0.80*)	(*0.87*)	(*0.80*)	(*4.77*)	(*3.92*)	(*0.07*)	(*0.36*)

*^a^Z-scores. ^b^Standard scores; other scores are raw scores. BD, bilingual dyslexics; MD, monolingual dyslexics; BC, bilingual controls; MC, monolingual controls.*

Having a formal diagnosis of developmental dyslexia based on standard criteria (ICD-10, World Health Organization [WHO], 2004) was the inclusion condition for the two dyslexic groups; all the diagnostic tasks were administered in Italian, which was the language of instruction for all the children.

Finally, no children had other diagnosed or referred cognitive deficits, hearing or vision disorders, nor comorbidity with other language disorders including developmental language disorder or specific language impairment. Children were recruited through contacts with the local health system (as for part of the dyslexic children) and through the schools they were in attendance at (as for the remaining dyslexic children and all the controls); no monetary compensation was provided to participants. The study was approved by the local Ethics Committee (Department of Neurological, Biomedicine and Movement Sciences, University of Verona, Verona, Italy) and conducted in accordance with the standards specified in the 2013 Declaration of Helsinki; moreover, written informed consent was given by the parents of all the children who took part in our research study.

### Materials

#### Preliminary Measures

All participants underwent a series of additional cognitive and linguistic tests. All children had to score within the normal ranges in the CPM Raven task measuring general intelligence (Raven et al., 1998; Italian standardization by Belacchi et al., 2008). Dyslexics had to score lower than −2SD below the mean of their reference category in two out of four reading measures (measured by speed and accuracy of word and nonword reading, *Batteria per la Valutazione della Dislessia e della Disortografia Evolutiva*, by Sartori et al., 2007). Conversely, typically developing children had to score within the normal ranges. We also assessed the children’s receptive vocabulary [by use of the *PPVT-R* by Dunn and Dunn (2000) Italian standardization by Stella, Pizzioli, and Tressoldi^6^], their WM (by administering the Forward and the Backward Digit Span task, FDS and BDS, of the WM test by Pickering and Gathercole, 2001) and their phonological competence [by administering a nonword repetition (NWR) task, see Vender et al. (under review)].

#### Modified Simon Task

The experiment was run on an Asus 15.6′ laptop using DMDX Automode version 4.3.0.1 software. The stimuli were four squares (dimensions 1012 × 536 pixels, BMP files) each for one of the four conditions. Each trial started with a fixation cross which appeared in the middle of the screen and remained visible for 500 ms and which was followed by a red or a blue square, either on the left or on the right side of the screen. As in traditional Simon tasks, participants were presented with four experimental conditions (blue congruent, blue incongruent, red congruent, and red incongruent) and instructed to press the number key 1 (on the left side of the keyboard) if they saw a red square and the number key 0 (on the right side of the keyboard) if they saw a blue square, irrespective of the position of the squares.

In our modification, the order of the colored squares presented to the subject was not random but instead determined by a simple deterministic recursive grammar; the Fib-grammar (described above). The strings of stimuli the grammar generates deliver a range of regularities: from simple local dependencies to higher order dependencies (as defined in section “The Fibonacci Grammar: A Simple Lindenmayer System”). From the subjects’ perspective the Simon task is unchanged; however, it is possible to track the subjects’ implicit learning of the regularities via RT and accuracy responses across the duration of the task.

Both accuracy and RTs were collected: each item remained on the screen for 1000 ms if there was no response before the next item was shown. Participants were asked to answer as quickly and accurately as possible. The timing started with the onset of the item and ended with the response of the subject. There were eight random practice trials in which subjects received feedback; after the training, they had the chance to ask questions before the experiment began. The modified Simon task comprised three blocks of 144 trials each, for a total of 432 stimuli, and took 10–15 min to complete.

As discussed in the introduction, the Fib-grammar comprises two rules, which converted into the colored stimuli are:

(5) red → blue (i.e., 0 → 1)
  blue → blue, red (i.e., 1 → 1, 0)

First of all, we wanted to verify whether there were improvements related to learning the following first-order transitional regularities:

(i) a red is always followed by a blue (a sequence of two reds is ungrammatical);
(ii) two blues must be followed by a red (a sequence of three blues is similarly ungrammatical), and
(iii) a blue can be followed by a red or a blue.

Moreover, in order to be sure that these improvements were related to the learning of these regularities and not to a general effect of habituation to the task, we compared performance in ambiguous (unpredictable) and unambiguous (predictable) items.

It must be noticed that, due to the formal properties of the grammar, as reviewed above, blue items were more frequent than red ones. Finally, as in every Simon task, both congruent and incongruent items were tested: unlike in traditional Simon tasks, however, the incongruent trial occurred every sixth item, for the reasons discussed above (see section “The Simon Task: Implications for Bilingualism and Dyslexia”).

To summarize, we employed this modified Simon task to identify differences in performance between monolingual and bilingual healthy and dyslexic children with the aim of assessing their ability to unconsciously pick up the regularities of the Fib-grammar.

First, we examined whether all groups successfully learned the regularities in (4a–c): the fact that a red is always followed by a blue was expected to be the easiest to acquire (section “Analysis 1: Blue Items Occurring After Red Items”). That two blues are followed by a red was instead predicted to be more difficult, since the memory load was higher: to succeed in this task, it is not sufficient to consider the item which has just appeared, but it is necessary to remember also the one occurring immediately before it (section “Analysis 2: Red Items Occurring After Two Blue Items”). Finally, to verify whether any improvements across blocks found in the previous analyses were really determined by the learning of the relevant regularities, and not by a general effect of habituation to the task, we compared RTs and accuracy in unambiguous trials (determined by 6a,b) and ambiguous ones (see 6c); lower or no improvement was expected in the ambiguous condition, where subjects could not benefit from learning the regularities delivered by the grammar (as discussed above) in predicting the color of the upcoming item (section “Analysis 3: Predictable vs. Unpredictable Items”).

### Procedure

All children were tested individually in a quiet room by the first author. They were administered the preliminary tasks followed by the modified Simon task. The Simon task lasted approximately 10–15 min, with a short break after the end of the second block. The whole experimental session lasted approximately 60 min (45 min for the preliminary tasks and 10–15 min for the Simon task).

## Results

### Preliminary Measures

Mean and SDs of each group in each preliminary task are reported in Table 2.

Results of the preliminary measures were analyzed by carrying out a series of one-way ANOVAs with group (MC, BC, MD, and BD) as the independent variable and performance in each task as a dependent variable. The four groups did not differ in age [*F*(3,104) = 0.720, *p* = 0.542] nor in general nonverbal intelligence [*F*(3,104) = 1.135, *p* = 0.339]. Conversely they differed in reading measures, including speed of word reading [*F*(3,104) = 33.249, *p* < 0.001], accuracy of word reading [*F*(3,104) = 39.335, *p* < 0.001], speed of nonword reading [*F*(3,104) = 28.830, *p* < 0.001], and accuracy of nonword reading [*F*(3,104) = 49.773, *p* < 0.001]. *Post hoc* comparisons with Bonferroni correction (*post hoc* comparisons henceforth) revealed that in word reading MD were slower than BC, MC (*p* < 0.001), and BD (*p* < 0.05), who were in turn slower than both BC and MC (*p* < 0.001); no differences were found between MC and BC (*p* = 1.000). Moreover, MD and BD were less accurate than BC and MC (*p* < 0.001); no differences were found between MD and BD (*p* = 0.939), neither between MC and BC (*p* = 1.000). As for nonwords, MD were slower than all other groups (*p* < 0.001), whereas BD were slower than BC (*p* < 0.01) and MC (*p* < 0.05). MC and BC performed similarly (*p* = 1.000); moreover, MD and BD were less accurate than MC and BC (*p* < 0.001); no differences were found between MD and BD (*p* = 1.000) and MC and BC (*p* = 1.000).

Differences were reported also in PPVT-R [*F*(3,104) = 11.163, *p* < 0.001]; as shown by *post hoc* comparisons, BD showed a poorer vocabulary in comparison to MD and MC (*p* < 0.001), whereas BC scored lower than MD (*p* < 0.01) and MC (*p* < 0.05). No differences were found between MD and MC (*p* = 1.000) and between BD and BC (*p* = 0.706).

Significant differences were also found for both FDS [*F*(3,104) = 6.593, *p* < 0.001] and BDS [*F*(3,104) = 5.624, *p* < 0.01]. *Post hoc* comparisons showed that in FDS, MD scored lower than MC (*p* < 0.01) but similarly to BC (*p* = 0.292), whereas BD scored lower than both MC (*p* < 0.001) and BC (*p* < 0.05). No differences were found between MD and BD (*p* = 1.000) nor between MC and BC (*p* = 1.000). As for BDS, instead, MD performed more poorly than MC (*p* < 0.05) and BC (*p* < 0.01), whereas BD had lower BDS scores than BC (*p* < 0.042) but not than MD (*p* < 0.292). No differences were found between MD and BD nor between MC and BC (*p* = 1.000).

Group differences were found also in NWR [*F*(3,104) = 34.680, *p* < 0.001]; as revealed by *post hoc* comparisons, both MD and BD performed worse than MC and BC (*p* < 0.001), whereas they performed similarly to each other (*p* = 0.327); also MC and BC performed similarly (*p* = 1.000).

Summarizing, the two dyslexic groups differed significantly from the control groups in all literacy measures, in WM tasks, and in phonological competence, whereas no differences were found in nonverbal intelligence and receptive vocabulary. The resulting profile is consistent with the typical cognitive and linguistic profile of children with dyslexia. Differences in vocabulary, but not in literacy, WM, and phonological competence are instead in line with the literature describing the typical profile of bilingual children (Bialystok et al., 2010). Since receptive vocabulary is reported to be relatively unimpaired in dyslexia (Vender et al., 2017), the fact that bilingual controls underperformed monolingual dyslexics and that no negative effects of dyslexia were observed should not be surprising.

### Modified Simon Task

In order to compare the performance of the four groups of children in the modified Simon task, both RTs and accuracy rates were considered. RTs were calculated only for correct answers, representing 93.59% of the responses. Answers given earlier than 200 ms, corresponding to 1.2% of the trials, were excluded from the analysis since they might reflect anticipatory response prior to proper stimulus processing. As outlined above, there was a time limit for participants’ responses, since the items disappeared after 1000 ms if no key was pressed; non-responses corresponded to 4.3% of the trials. All remaining trials were within the interval defined by the 2.5SDs intra-subject average, and thus no data were considered outliers. We then calculated the mean RT of each participant in each of the conditions tested.

In order to provide an answer to our research questions, aiming to verify whether participants showed evidence of having learnt the regularities of the input and whether group differences emerged, three distinct analyses were performed. In section “Analysis 1: Blue Items Occurring After Red Items,” the learning of the first regularity (a red is always followed by a blue) was investigated, whereas the fact that two blues are always followed by a red was assessed in section “Analysis 2: Red Items Occurring After Two Blue Items.” Finally, in section “Analysis 3: Predictable vs. Unpredictable Items,” we compared blue items being entirely predictable based on statistical regularities (blues occurring after a red) with those being completely unpredictable (blues occurring after a sequence of blue–blue–red–blue, which was ambiguous and could be followed by either a blue or a red, as discussed in section “The Fibonacci Grammar: A Simple Lindenmayer System”). This final analysis was particularly useful to verify whether improvements in the task were really dependent on the learning of the relevant regularities: if no differences between predictable and unpredictable trials were found, improvements could indeed be related to a general effect of habituation to the task, and not to implicit learning.

#### Analysis 1: Blue Items Occurring After Red Items

To verify whether children learnt that a blue item always appeared after a red one, we analyzed responses to all congruent and incongruent blue trials following a red one, comparing RTs and accuracy rates of the four groups of participants across the three blocks of stimuli. As shown in Tables 3, 4, reporting mean RTs and accuracy rates for each group in each block and condition, all groups displayed a decrease in RTs from Block 1 to Block 3, both in congruent and in incongruent trials. Moreover, bilingual dyslexics are faster than the other groups in each condition, whereas monolingual dyslexics were the slowest. As for accuracy, it was at ceiling for all groups in the congruent conditions, whereas it was lower in the incongruent trials, especially for the monolingual dyslexics.

**TABLE 3:** Mean (*standard deviation*) reaction times (RTs) in each condition for each group (“Analysis 1: Blue Items Occurring After Red Items”).

	**C_1**	**C_2**	**C_3**	**I_1**	**I_2**	**I_3**
BD	463.98	453.66	415.90	662.77	638.84	614.80
	(*73.47*)	(*62.76*)	(*70.89*)	(*85.30*)	(*80.96*)	(*95.61*)
MD	506.92	492.17	456.06	700.45	670.73	660.53
	(*71.91*)	(*67.06*)	(*62.41*)	(*82.47*)	(*110.16*)	(*97.25*)
BC	489.94	471.39	424.96	672.23	650.49	611.65
	(*65.58*)	(*64.90*)	(*60.17*)	(*83.88*)	(*105.10*)	(*112.70*)
MC	479.59	480.50	437.86	695.48	700.25	637.17
	(*75.08*)	(*79.61*)	(*70.97*)	(*93.29*)	(*95.09*)	(*91.45*)

*C, congruent; I, incongruent; 1, Block 1; 2, Block 2; 3, Block 3; BD, bilingual dyslexics; MD, monolingual dyslexics; BC, bilingual controls; MC, monolingual controls.*

**TABLE 4:** Mean (*standard deviation*) accuracy in each condition for each group (“Analysis 1: Blue Items Occurring After Red Items”).

	**C_1**	**C_2**	**C_3**	**I_1**	**I_2**	**I_3**
BD	0.97	0.96	0.97	0.79	0.79	0.82
	(*0.04*)	(*0.04*)	(*0.04*)	(*0.19*)	(*0.18*)	(*0.21*)
MD	0.97	0.92	0.96	0.77	0.71	0.80
	(*0.07*)	(*0.16*)	(*0.06*)	(*0.17*)	(*0.25*)	(*0.22*)
BC	0.99	0.97	0.98	0.79	0.80	0.81
	(*0.02*)	(*0.04*)	(*0.03*)	(*0.21*)	(*0.17*)	(*0.14*)
MC	0.99	0.99	0.99	0.88	0.92	0.92
	(*0.02*)	(*0.02*)	(*0.02*)	(*0.13*)	(*0.12*)	(*0.10*)

*C, congruent; I, incongruent; 1, Block 1; 2, Block 2; 3, Block 3; BD, bilingual dyslexics; MD, monolingual dyslexics; BC, bilingual controls; MC, monolingual controls.*

We ran a repeated-measures ANOVA with *Bilingualism* and *Dyslexia* as between-subject variables and *Congruency* (Congruent vs. Incongruent trials) and *Block* (1, 2, and 3) as within-subject variables.

As for RTs, we found a main effect of *Bilingualism* [*F*(1,104) = 5.521, *p* < 0.05, $\eta_{p}^{2}$ = 0.051], no main effect of *Dyslexia* [*F*(1,104) = 0.011, *p* = 0.916, $\eta_{p}^{2}$ = 0.000], and no *Bilingualism* × *Dyslexia* interaction [*F*(1,104) = 0.729, *p* = 0.395, $\eta_{p}^{2}$ = 0.007], indicating that bilinguals are faster than monolinguals in processing blue items occurring after a red one, irrespective of dyslexia. *Block* was also significant [*F*(1,104) = 43.415, *p* < 0.001, $\eta_{p}^{2}$ = 0.297], while the other interactions were not significant. This indicates that all groups showed an improvement in RTs across the task: specifically, RTs were faster in Block 2 than in Block 1 (*p* < 0.05) and in Block 3 than in Block 2 (*p* < 0.001). *Congruency* was also significant [*F*(1,104) = 966.322, *p* < 0.001, $\eta_{p}^{2}$ = 0.904], with congruent items being processed faster than incongruent ones. No interaction was significant, indicating that improvements were reported for both congruent and incongruent trials and for all groups.

As for accuracy, instead, we found a main effect of *Dyslexia* [*F*(1,104) = 11.047, *p* < 0.01, $\eta_{p}^{2}$ = 0.096], no main effect of *Bilingualism* [*F*(1,104) = 0.883, *p* = 0.350, $\eta_{p}^{2}$ = 0.008], and a significant *Bilingualism* × *Dyslexia* interaction [*F*(1,104) = 8.255, *p* < 0.01, $\eta_{p}^{2}$ = 0.074], indicating that the negative effect of dyslexia was limited to the monolingual children, with bilingual dyslexics performing more accurately than monolingual dyslexics and similarly to the two groups of controls. In this case, neither *Block* was significant [*F*(1,104) = 2.020, *p* = 0.135, $\eta_{p}^{2}$ = 0.019], nor the relevant interactions, indicating that no improvement in accuracy was found across the blocks in any of the groups.

*Congruency* was instead significant [*F*(1,104) = 192.397, *p* < 0.001, $\eta_{p}^{2}$ = 0.649], as well as the interaction *Congruency* × *Dyslexia* [*F*(1,104) = 3.920, *p* = 0.050, $\eta_{p}^{2}$ = 0.036] and the interaction *Congruency* × *Bilingualism* × *Dyslexia*: [*F*(1,104) = 7.181, *p* < 0.01, $\eta_{p}^{2}$ = 0.065], whereas *Congruency* × *Bilingualism* was not significant. To understand this interaction, we ran two separate two-way ANOVAs with *Bilingualism* and *Dyslexia* as fixed factors and mean RT in congruent trials or in incongruent trials as dependent variables. When considering congruent trials we found a significant effect of *Dyslexia* [*F*(1,104) = 9.449, *p* < 0.01, $\eta_{p}^{2}$ = 0.083], no effect of *Bilingualism* [*F*(1,104) = 0.181, *p* = 0.672, $\eta_{p}^{2}$ = 0.002], and no interaction between them [*F*(1,104) = 2.247, *p* = 0.137, $\eta_{p}^{2}$ = 0.021], whereas when considering incongruent trials we found a significant effect of *Dyslexia* [*F*(1,104) = 8.336, *p* < 0.01, $\eta_{p}^{2}$ = 0.074], no effect of *Bilingualism* [*F*(1,104) = 1.699, *p* = 0.195, $\eta_{p}^{2}$ = 0.016], but a significant interaction between them [*F*(1,104) = 8.627, *p* < 0.004, $\eta_{p}^{2}$ = 0.077], indicating that in incongruent trials monolingual dyslexics were less accurate than bilingual dyslexics, who performed similarly to the two control groups.

As these results show, all groups prove to have acquired the relevant regularity, showing increasingly lower RTs across the blocks. However, group differences were found: bilinguals were overall faster than monolinguals, and monolingual dyslexics were less accurate than the other groups, especially with incongruent items. Data point thus to the presence of a positive effect of bilingualism in dyslexia: bilingual dyslexics, indeed, were overall more accurate than their monolingual peers, and less disturbed by the presence of incongruent trials. The difference between monolingual and bilingual dyslexics was more evident in more complex conditions, in which higher processing costs are arguably required.

#### Analysis 2: Red Items Occurring After Two Blue Items

To assess the learning of the regularity predicting that two blues are always followed by a red and the presence of group differences, we considered all red items occurring after a sequence of two blues, comparing performance of the four groups across the three blocks, while distinguishing congruent and incongruent trials. Mean RTs and accuracy rates are reported in Tables 5, 6. In this case as well, all groups showed a decrease in RTs from Block 1 to Block 3; as in the previous analysis, monolingual dyslexics were the slowest, while bilinguals (both dyslexics and controls) were faster. All groups were more accurate in the congruent than in the incongruent conditions, with dyslexics being generally less accurate then controls.

**TABLE 5:** Mean (*standard deviation*) RTs (in ms) in each condition for each group (“Analysis 2: Red Items Occurring After Two Blue Items”).

	**C_1**	**C_2**	**C_3**	**I_1**	**I_2**	**I_3**
BD	528.16	546.49	499.90	673.46	686.13	669.20
	(*70.58*)	(*73.52*)	(*65.09*)	(*88.34*)	(*107.40*)	(*132.18*)
MD	569.47	566.88	534.42	708.33	701.03	703.35
	(*58.422*)	(*69.52*)	(*72.04*)	(*101.76*)	(*103.91*)	(*85.99*)
BC	531.51	532.75	491.25	634.39	634.70	609.51
	(*63.87*)	(*65.40*)	(*59.47*)	(*100.54*)	(*101.41*)	(*93.11*)
MC	551.39	553.10	512.23	646.68	682.91	620.45
	(*76.47*)	(*89.07*)	(*78.23*)	(*103.00*)	(*96.45*)	(*120.53*)

*C, congruent; I, incongruent; 1, Block 1; 2, Block 2; 3, Block 3; BD, bilingual dyslexics; MD, monolingual dyslexics; BC, bilingual controls; MC, monolingual controls.*

**TABLE 6:** Mean (*standard deviation*) accuracy in each condition for each group (“Analysis 2: Red Items Occurring After Two Blue Items”).

	**C_1**	**C_2**	**C_3**	**I_1**	**I_2**	**I_3**
BD	0.93	0.89	0.90	0.77	0.64	0.60
	(*0.05*)	(*0.07*)	(*0.11*)	(*0.16*)	(*0.28*)	(*0.27*)
MD	0.93	0.85	0.86	0.83	0.65	0.72
	(*0.07*)	(*0.17*)	(*0.11*)	(*0.17*)	(*0.30*)	(*0.25*)
BC	0.96	0.93	0.92	0.82	0.80	0.69
	(*0.07*)	(*0.07*)	(*0.08*)	(*0.21*)	(*0.23*)	(*0.29*)
MC	0.97	0.94	0.95	0.79	0.81	0.76
	(*0.05*)	(*0.07*)	(*0.06*)	(*0.23*)	(*0.18*)	(*0.22*)

*C, congruent; I, incongruent; 1, Block 1; 2, Block 2; 3, Block 3; BD, bilingual dyslexics; MD, monolingual dyslexics; BC, bilingual controls; MC, monolingual controls.*

As for RTs, we found a main effect of *Dyslexia* [*F*(1,104) = 3.863, *p* < 0.05, $\eta_{p}^{2}$ = 0.051], a marginally significant effect of *Bilingualism* [*F*(1,104) = 5.378, *p* = 0.052, $\eta_{p}^{2}$ = 0.037], and no interaction [*F*(1,104) = 0.083, *p* = 0.773, $\eta_{p}^{2}$ = 0.001], indicating that dyslexics were slower than controls, and that bilinguals tended to be faster than monolinguals.

*Congruency* was significant [*F*(1,104) = 561.869, *p* < 0.001, $\eta_{p}^{2}$ = 0.848], with incongruent items being processed more slowly than congruents. There was also a *Congruency* × *Dyslexia* interaction [*F*(1,104) = 12.947, *p* < 0.001, $\eta_{p}^{2}$ = 0.114], while the other interactions were not significant. Considering mean RTs in the whole task, we found that dyslexics were slower than controls with incongruent trials [*t*(106) = 3.156, *p* < 0.01], but not with congruent trials [*t*(106) = 0.966, *p* = 0.366].

*Block* was also significant [*F*(1,104) = 17.160, *p* < 0.001, $\eta_{p}^{2}$ = 0.145]; specifically significant differences were found between Block 2 and Block 3 (*p* < 0.001), but not between Block 1 and Block 2 (*p* = 0.543). No other significant interactions were found, indicating that improvements in RTs were equally reported in all groups and for both congruent and incongruent trials.

As for accuracy, we found a main effect of *Dyslexia* [*F*(1,104) = 8.249, *p* < 0.01, $\eta_{p}^{2}$ = 0.073], no effect of *Bilingualism* [*F*(1,104) = 0.619, *p* = 0.433, $\eta_{p}^{2}$ = 0.037], and no interaction [*F*(1,104) = 0.002, *p* = 0.961, $\eta_{p}^{2}$ = 0.000], indicating that dyslexics were less accurate than controls, irrespective of bilingualism.

*Congruency* was significant [*F*(1,104) = 159.283, *p* < 0.001, $\eta_{p}^{2}$ = 0.605], indicating lower accuracy for incongruent trials for all groups, as testified by the absence of significant interactions. *Block* was also significant [*F*(1,104) = 17.160, *p* < 0.001, $\eta_{p}^{2}$ = 0.145], as well as the interaction *Block* × *Dyslexia* [*F*(1,104) = 5.755, *p* = 0.004, $\eta_{p}^{2}$ = 0.052], *Congruency* × *Block* [*F*(1,104) = 3.779, *p* < 0.05, $\eta_{p}^{2}$ = 0.035], and *Congruency* × *Block* × *Dyslexia* [*F*(1,104) = 3.082, *p* < 0.05, $\eta_{p}^{2}$ = 0.029]. Paired sample *t*-tests separated for *Dyslexia* (dyslexics vs. controls) revealed that with congruent trials both groups showed a decrease in performance between Blocks 1 and 2 [dyslexics: *t*(47) = 3.451, *p* < 0.01; controls: *t*(59) = 3.809, *p* < 0.001], but not between Blocks 2 and 3 [dyslexics: *t*(47) = 0.556, *p* = 0.581; controls: *t*(59) = 0.527, *p* = 0.600]. As for incongruent trials, instead, dyslexics showed a decline between 1 and 2 [*t*(47) = 3.992, *p* < 0.001], and not between 2 and 3 [*t*(47) = 0.330, *p* = 0.743], whereas on the contrary controls showed a decline between Blocks 2 and 3 [*t*(59) = 2.576, *p* < 0.01], but not between Blocks 1 and 2 [*t*(59) = 0.061, *p* = 0.951]. This indicates that in the most complex condition (with the incongruent trials), dyslexics became inaccurate earlier than controls.

To sum up, all groups showed an improvement in RTs in correspondence to the red trials following a sequence of two blues, considering both congruent and incongruent trials. However, dyslexics were generally slower, especially with incongruent trials, whereas bilinguals tended to be faster. Concerning accuracy, instead, dyslexics made generally more errors than controls, irrespective of bilingualism, and all groups had more problems with incongruent stimuli. Moreover, accuracy decreased across the task, arguably as an effect of fatigue, especially for dyslexics who seem to be affected by tiredness earlier than controls.

#### Analysis 3: Predictable vs. Unpredictable Items

To verify whether the improvements in speed found across blocks in the previous analyses were really determined by the learning of the relevant regularities, and not by a general effect of habituation to the task, we compared RTs and accuracy of the four groups in predictable and unpredictable trials across the three blocks. For this purpose, we compared performance in items being unpredictable (where the blue trials followed a blue–blue–red–blue sequence and were thus uncontroversially ambiguous from the perspective of string-based statistical regularities, as discussed in section “The Fibonacci Grammar: A Simple Lindenmayer System”), and in the predictable items considered in section “Analysis 2: Red Items Occurring After Two Blue Items” (blue trials following a red). Since unpredictable items never occurred in correspondence to an incongruent trial, we considered only congruent items for this analysis. As can be noted in Tables 7, 8, responses to predictable items are generally faster and more accurate (ceiling performance) than those to ambiguous ones for all groups. As in the previous analysis, bilinguals are faster than monolinguals, irrespective of dyslexia, whereas both groups of dyslexics tend to be less accurate than controls.

**TABLE 7:** Mean (*standard deviation*) RTs (in ms) in each condition for each group (“Analysis 3: Predictable vs. Unpredictable Items”).

	**P_1**	**P_2**	**P_3**	**U_1**	**U_2**	**U_3**
BD	463.98	453.66	415.90	535.15	532.43	515.68
	(*73.47*)	(*62.76*)	(*70.89*)	(*79.67*)	(*87.42*)	(*92.70*)
MD	511.50	496.51	457.62	589.82	562.35	541.89
	(*69.86*)	(*65.01*)	(*63.33*)	(*79.33*)	(*74.74*)	(*84.80*)
BC	489.93	471.39	424.96	529.92	539.20	493.88
	(*65.58*)	(*64.90*)	(*60.17*)	(*68.08*)	(*72.97*)	(*59.83*)
MC	479.59	480.50	437.86	553.61	542.84	530.77
	(*75.08*)	(*79.61*)	(*70.97*)	(*93.12*)	(*92.39*)	(*92.04*)

*P, predictable; U, unpredictable; 1, Block 1; 2, Block 2; 3, Block 3; BD, bilingual dyslexics; MD, monolingual dyslexics; BC, bilingual controls; MC, monolingual controls.*

**TABLE 8:** Mean (*standard deviation*) accuracy in each condition for each group (“Analysis 3: Predictable vs. Unpredictable Items”).

	**P_1**	**P_2**	**P_3**	**U_1**	**U_2**	**U_3**
BD	0.97	0.96	0.97	0.92	0.94	0.89
	(*0.04*)	(*0.04*)	(*0.04*)	(*0.10*)	(*0.07*)	(*0.13*)
MD	0.96	0.92	0.96	0.92	0.83	0.90
	(*0.07*)	(*0.16*)	(*0.06*)	(*0.10*)	(*0.25*)	(*0.13*)
BC	0.99	0.97	0.98	0.96	0.94	0.94
	(*0.01*)	(*0.04*)	(*0.03*)	(*0.09*)	(*0.08*)	(*0.10*)
MC	0.99	0.99	0.99	0.97	0.96	0.95
	(*0.02*)	(*0.02*)	(*0.02*)	(*0.04*)	(*0.06*)	(*0.08*)

*P, predictable; U, unpredictable; 1, Block 1; 2, Block 2; 3, Block 3; BD, bilingual dyslexics; MD, monolingual dyslexics; BC, bilingual controls; MC, monolingual controls.*

We ran a repeated-measures ANOVA with *Bilingualism* and *Dyslexia* as between-subject variables and *Predictability* (Predictable vs. Unpredictable) and *Block* (1, 2, and 3) as within-subject variables.

As for RTs, we found a main effect of *Bilingualism* [*F*(1,104) = 4.765, *p* < 0.05, $\eta_{p}^{2}$ = 0.044], no main effect of *Dyslexia* [*F*(1,104) = 0.488, *p* = 0.486, $\eta_{p}^{2}$ = 0.005], and no *Bilingualism* × *Dyslexia* interaction [*F*(1,104) = 1.308, *p* = 0.255, $\eta_{p}^{2}$ = 0.013], indicating that bilinguals are generally faster than monolinguals, irrespective of dyslexia.

*Predictability* was also significant [*F*(1,104) = 236.710, *p* < 0.001, $\eta_{p}^{2}$ = 0.697], with predictable items yielding faster RTs than unpredictable ones. This held for all groups, as testified by the absence of significant interactions. *Block* was also significant [*F*(1,104) = 41.946, *p* < 0.001, $\eta_{p}^{2}$ = 0.289], while the other interactions were not significant. This indicates that all groups showed an improvement in RTs across the task. *Predictability* × *Block* was also significant [*F*(1,104) = 5.306, *p* < 0.01, $\eta_{p}^{2}$ = 0.049], while the other interactions were not. Paired samples *t*-tests revealed a significant improvement in RTs from Block 1 to Block 2 for predictable items [*t*(107) = 2.248, *p* < 0.05] but not for unpredictables [*t*(106) = 1.048, *p* = 0.297]; both unpredictable and predictable items, instead, were processed faster in Block 3 than in Block 2 [respectively, *t*(107) = 9.428, *p* < 0.001; and *t*(106) = 3.461, *p* < 0.01].

Regarding accuracy, instead, which was overall very high for all groups and especially for predictable items, we found a main effect of *Dyslexia* [*F*(1,104) = 10.801, *p* < 0.01, $\eta_{p}^{2}$ = 0.094], no main effect of *Bilingualism* [*F*(1,104) = 0.238, *p* = 0.627, $\eta_{p}^{2}$ = 0.002], and no *Bilingualism* × *Dyslexia* interaction [*F*(1,104) = 2.465, *p* = 0.120, $\eta_{p}^{2}$ = 0.023], indicating that dyslexics were less accurate than controls.

*Predictability* was significant [*F*(1,104) = 54.813, *p* < 0.001, $\eta_{p}^{2}$ = 0.345], but not its interactions, indicating that predictable items were processed more accurately (with almost 100% accuracy for all groups) than unpredictable ones by all groups.

*Block* was also significant [*F*(1,104) = 3.671, *p* < 0.05, $\eta_{p}^{2}$ = 0.034], as well as the interaction *Block* × *Bilingualism* × *Dyslexia* [*F*(1,104) = 5.123, *p* < 0.01, $\eta_{p}^{2}$ = 0.047]. No other interaction was significant. To understand the interaction, we ran a series of paired samples *t*-tests comparing general accuracy in Blocks 1, 2, and 3 in all four groups. We found that monolingual dyslexics performed worse in Block 1 than in Block 2 [*t*(23) = 2.281, *p* < 0.05], but similarly in Blocks 2 and 3 [*t*(23) = 1.484, *p* = 0.151]. Bilingual dyslexics showed instead the opposite trend, performing similarly in Blocks 1 and 2 [*t*(23) = 0.893, *p* = 0.381], but worse in Block 3 than in Block 2 [*t*(23) = 2.095, *p* < 0.05]. The two groups of controls showed instead a similar performance in all blocks [bilingual controls, Blocks 1–2: *t*(29) = 1.818, *p* = 0.079, Blocks 2–3: *t*(29) = 0.367, *p* = 0.716; monolingual controls, Blocks 1–2: *t*(29) = 0.650, *p* = 0.521, Blocks 2–3: *t*(29) = 0.771, *p* = 0.447]. This seems to indicate that, although both groups of dyslexics become generally more inaccurate throughout the task, which could be again an effect of fatigue, monolingual dyslexics seemed to be affected by tiredness earlier than bilingual dyslexics.

Summarizing, results show that, although RTs decreased for both predictable and unpredictable items, the improvement was significantly higher for the predictable items, indicating that it must be due to the learning of the relevant rules. This was also confirmed by the fact that accuracy was higher in predictable items. Notice moreover that the absence of interactions with predictability indicates that group differences, with bilinguals being faster and dyslexics being less accurate, held for both cases.

## Discussion

In this study, we assessed learning of an artificial grammar in monolingual and bilingual children, with and without a diagnosis of dyslexia, by means of a modified Simon task in which the order of the stimuli was not random but determined by the Fibonacci grammar.

As emphasized in section “Research Questions and Predictions,” we were interested in investigating (i) whether there was implicit learning of the regularities characterizing the Fibonacci grammar and (ii) whether group differences emerged, especially in relation to the interaction between bilingualism and dyslexia. To address these research questions, we ran three separate analysis, comparing the performance of the four groups in learning that a red is always followed by a blue (section “Analysis 1: Blue Items Occurring After Red Items”) and that two blues are always followed by a blue (section “Analysis 2: Red Items Occurring After Two Blue Items”). To be sure that improvements were really related to the learning of these statistical regularities, and not to a general effect of habituation to the task, we also compared the blues following a red, which were completely predictable, to the blues following the sequence of blue–blue–red–blue, which were instead unpredictable (section “Analysis 3: Predictable vs. Unpredictable Items”).

Although group differences were found, with bilinguals being always faster than monolinguals and dyslexics less accurate than controls, as will be discussed below, it is worth emphasizing that all groups showed evidence of implicit learning, as clearly confirmed by shorter RTs and improved accuracy found in unambiguous trials, which could be correctly foreseen once these regularities were learnt. In ambiguous trials, instead, the impossibility to rely on local transition probabilities prevented participants to perform as fast and accurately as with the predictable ones. Although RTs decreased for both types of trial, as a possible effect of habituation to the task, we found that the improvements in RTs and accuracy were significantly higher for the unambiguous trials, suggesting that learning had occurred. Moreover, improvements in unambiguous trials were found as early as between Blocks 1 and 2, but only between Blocks 1 and 3 for ambiguous trials. This indicates that the learning of the regularities yielded by the Fibonacci grammar took place relatively early and, in fact, before the appearance of the habituation effect to the Simon task. Finally, group effects were similar across ambiguous and unambiguous trials, with bilinguals exhibiting faster RTs and dyslexics lower accuracy.

Given these general learning effects, we further verified whether each of the two first-order transitional regularities [see 4(a–c)] had been learnt. According to the first regularity, red trials could only be followed by blue ones: results confirmed that this regularity was successfully acquired by all groups, as showed by increasingly shorter RTs, with differences being detected as early as between Blocks 1 and 2. Importantly, this improvement was found for both congruent and incongruent trials, with responses to the latter being slower and less accurate. As for accuracy, we found a negative effect of dyslexia limited to the monolingual children: specifically, bilingual dyslexics were more accurate in reacting to incongruent trials than monolingual dyslexics, and as accurate as the two control groups. This suggests that bilingualism could confer an advantage to the impaired children in the most difficult experimental conditions.

We observed that learning also took place for the second regularity, according to which a sequence of two blues must be followed by a red: again, this was observed in both congruent and incongruent trials for all groups, who showed decreased RTs between Blocks 2 and 3, suggesting that this regularity was acquired at a later stage than the first one. This is arguably related to its higher complexity, which requires participants to consider not only the immediate predecessor of the current stimulus, but also the preceding one. In this case as well, group differences were found; dyslexics were slower, especially in incongruent trials, and also less accurate than controls, whereas bilinguals tended to be faster than monolinguals. In this case, we also found a decrease in accuracy: all groups, despite being faster in predicting the occurrence of a red trial after two blues, became less accurate as the task progressed. This is arguably an effect of fatigue, particularly evident in this more difficult condition.

Summarizing, our findings lead to the important conclusion that all groups of subjects, including the children suffering from dyslexia, were able to learn the first-order regularities characterizing the Fibonacci grammar used, generated as a specific instantiation of a Lindenmayer system and assessed by means of a modified Simon task.

The other crucial focus of our work lied in the analysis of the effects of bilingualism and dyslexia in this task: interestingly, we found that bilinguals, both dyslexics and controls, were always faster than monolinguals in reacting to the stimuli appearing on the screen, for both congruent and incongruent trials. This points to a generalized bilingual advantage, consistently with other studies reviewed in the introduction and reporting shorter response times by bilinguals in the Simon task. Importantly, our results point to an extension of the advantages of bilingualism also to impaired children, indicating that bilingualism could be beneficial for dyslexics, who in some cases even performed at the level of the monolingual controls (Analysis 2: Red Items Occurring After Two Blue Items), at least in the domain of EFs and controlled attention. Conversely, dyslexics, including both monolinguals and bilinguals, were generally less accurate than controls, indicating that they struggled more than their peers with the Simon task. This result is in line with our expectations too: as argued in the literature and discussed above dyslexia can also be characterized in terms of a processing inefficiency, leading to reduced processing and memory resources available to impaired children, as well as to lower levels of controlled attention and interference suppression. This is also compatible with the fact that poorer responses were more marked in the presence of items requiring more complex processing (incongruent trials) and thus, arguably, more effortful to learn. These results confirm our expectations about group differences in the task, with dyslexics showing difficulties arguably due to their processing or memory limitations. Bilinguals, on the contrary, displayed an advantage over monolinguals which, interestingly, extended to impaired subjects, and which could be interpreted as reflecting bilinguals’ increased abilities in tasks requiring controlled attention.

To sum up, our results prompt two interesting considerations, related to the novelty of our protocol and to our research questions. First, on the one side, we extended the results that have been obtained with grammars traditionally employed in the AGL literature. Our results show that learning of an artificial grammar takes place even with a generative system that instantiates more abstract, and relatively language-independent, grammatical knowledge. On the other side, we demonstrated that learning of grammar-induced regularities can be detected with a modified Simon task, which has the advantage, of maximizing the elimination of residual explicit learning and metarepresentational awareness effects that are often found in AGL investigation. More particularly, in such SRT paradigms, the subjects are never explicitly made aware of being involved in potential grammatical learning. Firstly, they are distracted from paying attention to the statistical regularities in the succession of the visual stimuli, since they have to cope with the cognitive challenge represented by the potential asymmetry of location between visual stimulus and motor response. Secondly, as is generally the case for SRT tasks, subjects are never asked about the potential learning outcome, which could be objectively detected, in our protocol, in terms of increased reduction of RT for the predictable trials with respect to the unpredictable ones, besides the generalized RT reduction that can be interpreted as an effect of habituation to the task. Therefore, our results convincingly show that the observed learning must have taken place implicitly, while subjects were focused on an entirely different task (correctly reacting to blue and red squares irrespective of the location at which they appear on the screen) and are therefore throughout the whole process unaware of analyzing potential regularities in the sequence of items.

Second, as for the existence of group differences our data point to a general bilingual advantage in terms of RTs and to a general dyslexic disadvantage in terms of accuracy in the task. As discussed above, the shorter RTs of bilinguals can be attributed to their enhanced attentional control and specifically to their ability to maintain high levels of attention in performing the task, whereas the difficulties exhibited by dyslexics can arguably be attributed to their lower processing resources. Crucially, the bilingual advantage has also been found in impaired children: bilingual dyslexics consistently performed better than the monolingual dyslexics, reaching the accuracy levels of the two control groups in the acquisition of the easiest regularity (predicting that a red is always followed by a blue). This result suggests that bilingualism does not produce negative effects in dyslexics, as is sometimes erroneously believed; on the contrary, it can lead to significant cognitive and linguistic advantages.

Finally, this bilingual advantage is found in the familiar domain of attentional control and inhibitory skills and cannot easily be directly attributed to enhanced performance at the level of implicit learning. As repeatedly emphasized, our results show that implicit learning took place for all groups involved, crucially including (monolingual) dyslexics. In fact, as a measure of methodological caution, it must be acknowledged that all group differences we detected concerned both ambiguous and unambiguous trials, to the effect that it is difficult to disentangle the cognitive effects induced by the Simon task from those linked to the implicit learning task. We leave this issue to future research. A natural follow up could be that of administering subjects, besides our modified Simon task, a traditional Simon task, in which the sequence of the items is really random, in order to evaluate the emergence of group differences based on direct comparison between the measurement of group effects in implicit learning and the measurement of group effects in EF enhancement.

Another exciting direction of development aims at disentangling the effects of implicit learning that may be exclusively rooted in the computation of statistically based transitional probabilities from the (possible) effects that stem from the subject’s capacity to assign a hierarchical structure, given the sequences generated by the Fib-grammar. As discussed in the introduction the latter is a necessary condition that must be met in order to perform above-chance in the choice of the following symbol when presented with a sequence *blue–red–blue–red–blue* (i.e., 10101). These local configurations differ in constituency structure with respect to the local sequence *blue–blue–red–blue* (i.e., 1101), which we have used in the present study to define string-based *real* points of *ambiguity* [see Krivochen et al. (2018) for formal discussion].

In this way, the methodological advantages of our modified Simon task could be made relevant not only for measuring and evaluating learning differences among populations, but also for assessing the precise nature of implicit learning and discriminating between different accounts of implicit learning.

## Conclusion

In this experiment, implicit learning of an artificial grammar in monolingual and bilingual children with and without dyslexia was investigated by means of a modified Simon task (a specific instance of SRT task) in which the sequence of stimuli followed the rules of a Fib-grammar (one of the Lindenmayer systems). Results clearly support the idea that learning took place, since participants of all groups became increasingly sensitive to properties of the input manifested by local sequences of red and blue items. Importantly, the two low-level regularities that we assessed [in (4a–b); i.e., a red is followed by a blue, and two blues are followed by a red] were acquired by all groups; however, overall group differences were found, with bilinguals being faster than monolinguals, and dyslexics less accurate than controls. These results, besides pointing toward some new exciting avenues of research, as discussed above, already clearly indicate that the benefits of bilingualism crucially extend to impaired children, suggesting that bilingualism should be encouraged and supported also in linguistically impaired individuals.

## Data Availability

The datasets generated for this study are available on request to the corresponding author.

## Ethics Statement

The study was approved by the local Ethics Committee (Comitato Etico del Dipartimento di Scienze Neurologiche e del Movimento dell’Università degli Studi di Verona; “Ethic Committee of the Department of Neurological and Movement Sciences at the University of Verona”) and conducted in accordance with the standards specified in the 2013 Declaration of Helsinki.

## Author Contributions

DS conceived and developed the modified Simon task. DD and MV conceived the whole experimental protocol. MV collected the data, ran the statistical analyses, and wrote the manuscript. DK and BP contributed to the writing of section “The Fibonacci Grammar: A Simple Lindenmayer System”. All authors contributed to the interpretation of the results, revised the work critically for important intellectual content, and gave the final approval of the version to be published.

## Conflict of Interest Statement

The authors declare that the research was conducted in the absence of any commercial or financial relationships that could be construed as a potential conflict of interest.

## Footnotes

1 Developmental dyslexia is a genetic disorder characterized by a difficulty in properly acquiring reading and spelling skills, despite adequate classroom exposure, in absence of cognitive, physical, or sensorimotor impairments and socio-economical or emotional problems (Vellutino, 1979). Beyond literacy problems, dyslexia is characterized by marked linguistic deficits, affecting in particular phonological, morphological, and grammatical competence, as well as by WM and processing deficits (Vender, 2017).

2 The Fibonacci grammar owes its name to the number of total items generated per row as well as of 0s and 1s individually, which follows the Fibonacci sequence (1, 1, 3, 5, 8, 13, …); see Figure 1.

3 This is not surprising if we consider that the Fibonacci sequence itself is defined as a recurrence relation, where for any term F_*n*_ we have that:

(i) F_*n*_ = F_*n*–1_ + F_*n*–2_

Analogously, any generation G_*n*_ can be defined by the following formula:

(ii) G_*n*_ = G_*n*–1_^∧^ G_*n*–2_

where ^∧^ indicates concatenation. Note that, because the relation ^∧^ is not commutative (i.e., does not produce an identical output regardless of the order of items, unlike the operation +), left-concatenating generation G_*n*–1_ to G_*n*–2_ does not yield the same result as right-concatenating G_*n*–1_ to G_*n*–2_ (Krivochen and Phillips, 2018). This is a non-trivial property which is essential to be aware of in order to make predictions about the symbols that come up in the string at any juncture.

4 Due to the difficulties of recruiting a sufficient large sample of bilingual dyslexic children speaking the same L1, we included in our sample children with heterogeneous L1s. The L1s of the BD were: Albanian (seven children), Arabic (six), Romanian (five), Spanish (two), Hindi (one), Turkish (one), Yoruba (one), and Senegalese Wolof (one). The L1s of the BC were: Romanian (nine children), Arabic (eight), Albanian (four), Hindi (two), Spanish (one), Ghanaian English (one), Yoruba (one), Moldovan (one), Serbian (one), Polish (one), and Macedonian (one).

5 Information about the exposure to the two languages were collected by administering the Bilingual Language Exposure Questionnaire [see Unsworth et al. (2012) and Vender et al. (2016) for a description of the task and for the discussion of the concepts of TLE, a traditional index calculating subtracting the AFE of the child to their chronological age, and CLE, which is a more precise measure considering the different exposures to the two languages of the child in both the present and the past].

6 PPVT-R is a task addressing receptive vocabulary; children are asked to point at a picture out of an array of four alternatives to select the one that represents the word uttered by the experimenter.
